# Essays on Medical Education, and the Medical Profession in the United States

**Published:** 1831

**Authors:** 


### WESTERN JOURNAL

OF THE

### MEDICAL AND PHYSICAL SCIENCES.

JULY, AUGUST, AND SEPTEMBER, 1831.

### Essays and Cases.

Art. I.—*Essays on Medical Education, and the Medical Profession in the United States.*—By the Senior Editor.

### ESSAY IV.

### STUDIES, DUTIES, AND INTERESTS OF YOUNG PHYSICIANS.

A diploma constitutes the great object of ambition with every student of medicine. This being acquired, he feels disposed to relax in his efforts; and enjoy his new dignity. In the opinion of his teachers, and, of course, of himself, he is qualified for the practical duties of the profession; and a happy feeling of self-complacency, sometimes of self-sufficiency, springs up. He is no longer a pupil, but a physician—walks on a higher level—is surrounded by new associates—and either turns his thoughts towards new objects, or to none at all.

The length of this period, varies with the temper and necessities of each individual. If voluntarily prolonged, the omen is decidedly bad. It indicates a want of ambition, of industry, of conscientiousness, or of the *amor scientiæ*. I am grieved to say, that too many of our young physicians, are deficient in one or more of these important elements of future eminence; an through life, prefer to vegetate in the lower walks of the profession, when they might ascend and flourish in its higher regions.

The young man of merit, regards a diploma, as only one of the series of honors, which lie within his grasp. He stops to breathe, but still remembers that he has passed through his novitiate only, and must speedily resume the labors by which alone he can hope to reach the highest honors of the profession. Many desire, even resolve, to be distinguished, but neglect to employ the proper means. Aware of the necessity of study, they intend to recommence it; but put off the day of renewed labor, till it is too late. Habits of idleness are formed; the allurements of society establish their fascination; examples of partial or temporary success in the acquisition of *business*, without an attention to studies, unfortuately surround and encourage them; the ignorance and credulity of the world, disclose to them the possibility of acquiring, without deserving its confidence; hard study begins to appear less indispensable, and more repulsive; their noble resolves are forgotten—their destiny becomes fixed, and a humble one it is. Such are the course and termination, of a majority of those who are put to the study of medicine, in the United States.

The physician who neglects his studies for the few first years after graduation, will seldom resume them. In many respects, this is one of the most important periods of his whole life. The omissions of youth may be supplied—those of manhood are fatal to our prospects, in proportion to their number and magnitude.

The young physician is not aware how soon his elementary knowledge—much of which is historical and descriptive, rather than philosophical—will fade from his mind, when he ceases to study. That which he possesses can only be retained by new additions. He cannot remain stationary; the moment he ceases to acquire, he loses; and this is true, not merely of professional, but of classical and general knowledge, as I have warned him in a previous essay.

Hence, every young physician, should devote a stated portion of time to the branches of learning which engaged (or should have engaged) his attention, before he began the study of medicine. By doing this, he would, if previously well taught, soon make himself an able and accurate scholar; or, having been denied the advantage of early opportunities, he might supply many deficiencies, and save himself from the odium of gross illiterateness. It is *this* ignorance, still more than defective professional attainment, that constitutes the difference between the physicians of America and Europe. It perpetuates itself, because the *majority* of us, being in the same condition, are disposed, mutually, to sustain each other against the contempt of sound scholarship.

It is equally necessary for the young physician to review his professional studies, especially his anatomy. Should *it* once glide from him, the substratum of his surgery, physiology, and pathology, is gone. To prevent this, he should study it in connexion with these important branches, and unite the whole into one system; by which only, can it be retained, or they illustrated. But he must not merely review, he must *extend* his professional studies. Previous to graduation, the most industrious student can study but a small part of the books, which are current in the profession. He has had before him but a few specimens in each department: a large portion remains to be studied. In this duty he should proceed with method; and I will mention two principles which should govern him:—First, he ought successively to undertake the study of particular diseases, or groups of diseases, and seek, in all the books consecutively, for what relates to them. In this manner, he will form his own monographs. Secondly, when actual cases of disease present themselves, he should read whatever may be within his reach in reference to them. Such readings are more profitable than any others, because the motives which prompt him, sustain his attention and invigorate his understanding.

The same is the appropriate period, for the study of retrospective medical literature. But few of the works of past ages should be put into the hands of an under graduate; but no physician should regard his education as in any degree complete, until he has read the writings of most of the great observers, and original geniuses, who have adorned the profession in different ages. He will thus trace up established principles, through all their modifications, to their sources, perhaps in a remote antiquity. He will, at every step, amass important but neglected facts. He will be taught how to think. Finally, his mind will be enlarged and liberalized, by an acquaintance with the revolutions which the science has undergone.

The auxiliary sciences should claim the attention of the young physician. The most important of these, are, comparative anatomy, zoology, botany, mineralogy, and meteorology; all of which, he will soon discover, have many interesting relations with the profession. With the utmost assiduity, he cannot, in the course of a three years' medical pupilage, acquire even the rudiments of these collateral sciences; and if he postpone the study of them till he has acquired professional business, he will have but little time to devote to their elements, although many opportunities must offer in the midst of business for reducing such knowledge to practice.

I would be superfluous, to say more on the subjects of study, in the first years of professional life. To regard that period as one of admissible leisure, argues either a pitiable ignorance of the extended limits of the medical profession, or a culpable disregard for its weighty and dignified responsibilities.

This is especially true, of country practitioners; who will find their time on distant visits, profitably occupied, in observing and collecting the natural productions of the district of country in which they reside; and, over which, most of them continue to travel for years, in utter ignorance of the interesting objects which it presents. By a very moderate attention to botany and mineralogy, the country physician, without the smallest neglect of his professional duties, would, in a short time, become well acquainted with the plants and minerals of his vicinity; and might thus make important contributions to the natural history of his country, while the *tœdium* of his solitary rides would be effectually obviated.

The young physician should write as well as read. He should study with his common-place book by his side, especially when engaged in works which he does not expect to peruse a second time—and make to them such references, or, from them such extracts, or, of them such abridgments, as may seem advantageous. It is a most useful exercise, to abridge and rewrite such parts of the greater authors, as relate to some interesting subject, and place them in *juxta position*, for the purpose of comparison or contrast. He should sometimes epitomize entire works, for the mere value of the exercise in professional composition; and to the same end translate, largely, from all the languages he may cultivate. He ought carefully to record his own observations and experiments; not in a diffuse and inaccurate style, but with precision and perspicuity. He should from time to time write essays and dissertations on speculative topics, and at subsequent periods subject them to a rigid criticism. By these various exercises, he will, at length, acquire a habit of expressing himself with facility and clearness, if he should never attain to elegance; while these repeated attempts at arranging and connecting facts, will have strengthened his reasoning powers, and given him habits of method. It is lamentable to see how few of us write well, that is, with simplicity, conciseness, and purity. The fault lies quite as much in neglect of practice in composition, as in the want of previous education. Neither will do alone; but of the two, the former is, perhaps, the more important.

Young physicians should write much, but publish little. Every good composition is not worthy of being read. It is easy to make new digests of common facts, in the form of essays and dissertations; or to report new cases, which differ in no essential point from those with which the books are already crowded. Such contributions swell, without enriching the archives of the profession. Every new arrangement of facts, should establish or subvert some principle, or rule of practice; and every reported case, should present, at least, one point of novelty. In reference to style, the *desideratum* of the former, is method—of the latter, brevity; and without these requisites, neither will be read with pleasure, whatever may be the value of its materials.

Inexperienced physicians should avail themselves of every opportunity, to reduce their theoretical knowledge to practice. They must never wait for select cases, nor shrink from a professional duty, however repulsive. Every young physician must expect to begin his career among the poor. Society attaches great importance to experience; and those who can command the services of one who has had practice, will not confide in a novice, whatever may be his genius or attainments.

The epoch of professional life now under consideration, is the time to perfect those habits of observation, which begin in our pupilage. Every clinical case ought to be a study, and nothing connected with it should be overlooked. I never knew a skilful practitioner who was not an acute observer. Whatever may be a physician's judgment, he should not be confided in, if obtuse in his powers of observation. To arrive at correct results, the first and greatest requisite, is, to have correct *data*, which can only be acquired by accurate and patient observation.

It is a prevailing opinion, that a physician should marry young. The beneficial influence of this step on the business of a young physician, is, I think, overrated. I have seen many acquire a respectable practice before marriage; and others fail in that particular, although they married early. A compliance with the maxim has, moreover, often led to a premature settlement of this kind, and done irreparable injury to the individual, by augmenting his expenses beyond his slender income; thus multiplying his cares and anxieties, so as greatly to abridge his hours of professional study. On the whole, however, it is necessary and proper for a physician to marry, as early as prudence will justify; for, to a certain extent, it *does* promote the acquisition of business; while it prevents the formation of habits of *solitary* study, which he cannot afterwards change. I look upon such habits, in a country where the domestic relations are so generally cherished as in the United States, as exceedingly detrimental to the cultivation of the profession. The physician who cannot study in the bosom of his family, and amidst the interruptions produced by domestic duties and professional labors, must neglect either one or the other. Early marriage, before the establishment of fixed habits of secluded study, will, in general, save a professional man from this unfortunate dilemma. If he begin, in due time, to read and write, at the family fire-side, he will daily devote many hours, to both, that would otherwise be entirely lost; and thus be made, not only a better physician, but a better husband and father.

The same period of life, is that in which a physician is to form his manners, or rather his *manner*. In no vocation is manner more influential. Many young physicians have acquired popularity, by the mere force of manner, while others, of equal or greater professional skill, from the same cause, have never become favorites with the public. For such cases I can offer no infallible *recipe*. Dignity, tenderness, and modesty, would seem to be appropriate elements in the deportment of a young physician, whatever may be their effect on the people. They may not inspire confidence in the vulgar, in comparison with impudence and garrulity; but, sooner or later, their influence will be felt and acknowledged. It must, however, be admitted, that public favor is not always the meed of unexceptionable manners, even when sustained by competent skill.

In these instances, however, there is, generally, in the midst of prevailing excellences, some particular defect. For example, the individual may be deficient in *tact;* or wanting in attention and earnestness—a defect, which, more than most others, will retard the acquisition of business. Nothing captivates the friends of a sick person so much, as an earnest and anxious manner on the part of the physician. None are insensible to such a manner, while many will receive it as a substitute both for skill and social refinement.

But of all the extraneous aids to the acquisition of business, some *peculiarity* of manner is, perhaps, the most efficient. Should it even border on the absurd or the ridiculous, it is still in the opinion of the people, an indication of genius, and seldom fails to secure their confidence. To be effective, however, it must be natural, not assumed, as affectation is with difficulty concealed, and its discovery of course, brings contempt.

One of the first questions which perplexes a young man after his graduation, is the choice of a residence. He should, if possible, place himself within the pale of good, that is, of intelligent, society. In any other situation, both his mind and manners will degenerate. His companionship should be with the enlightened and refined of both sexes; for his character is to be formed, and should be modelled after good examples. It is not important that he should remain permanently in his first locality. He should not leave it, however, till he has passed through a period of probation, and acquired a competent amount of practical knowledge. He may then remove to a more extended theatre of action, where he ought to spend his life. If he have commenced within the precincts of such a situation, he should hold on till he acquires public confidence, however slow it may seem to be in coming. Not unfrequently this is a protracted period, and abounds in anxieties, hopes and fears, impatience, and hypochondriacism. Under these feelings, the young physician too often resolves on a change of residence; and, at the moment, when he is on the eve of acquiring business, transfers himself to another town, where, being a stranger, he is doomed to a second probation. Such removals, several times repeated, have proved fatal to the prospects of many, who, remaining patiently in one situation, would have found it productive of all they could desire.

Nearly connected in its nature and consequences, with this subject, is an attention to some other business, than that which is professional. In some instances the young physician relinquishes his profession, determining to resume it after a time; but, oftener, he attaches to it, some other pursuit, which is, generally, that of an apothecary. Both experiments are dangerous, and too often lead to the destruction of all professional prospects. It is far better for the young practitioner, however straitened may be his circumstances, to struggle on, with professional distinction exclusively in view; and the attention of the public directed to him as a physician and surgeon only. Society is not disposed to admire or regard a man, in more than one character; and all who are candidates for public patronage, should present themselves in the character which they are most anxious to sustain. A young physician, moreover, is, in general, not qualified for any business but that of his profession; and incurs the risk of pecuniary embarrassments, whenever he attempts a vocation, to which he has not been educated. And this leads me to warn the young physician against contracting debts and liabilities. The young merchant may contract debts with propriety and advantage. His character for punctuality is a part of his capital, and should be used; but the only capital of a young physician is his reputation for professional learning, diligence, and fidelity. On this character he should place his sole reliance. All his efforts should be directed in its establishment; and whatever can interfere, with this important and difficult object, should be studiously avoided.

He who relies on his earnings for support, will be led, not only to practice economy, but to keep his accounts with accuracy, and to attend carefully to the collection of his debts,—habits that will exert a beneficial influence on his character and happiness throughout life.

I am aware, that much of what is contained in this essay, will be regarded as common place; but I cannot admit, that it is not ultimately connected with the dignity of the profession, and the prosperity of its members. The former, indeed, can only result from the latter. Hence all who desire the advancement of the profession, should labor to render those who practice it, respectable in all their relations to society. In proportion as this is accomplished, will the profession exert that influence to which it may justly aspire.

